# Atraumatic Isolated Stress Fracture of the Distal Tibial Metaphysis in a Pre-menopausal Patient: A Case Report

**DOI:** 10.7759/cureus.34068

**Published:** 2023-01-22

**Authors:** Mohammad Daher, Dany Aouad, Rabih Kortbawi, Rami Ayoubi, Chawki Kortbawi

**Affiliations:** 1 Orthopedics, Hotel dieu de france, Beirut, LBN; 2 Orthopedics, Saint George University Hospital Medical Center, Beirut, LBN

**Keywords:** tibial metaphysis, pre-menopausal, distal tibia, atraumatic, stress fractures

## Abstract

Stress fractures are partial or complete bone fractures usually occurring in the weight-bearing bones resulting from repeated cycles of submaximal stress and bone remodeling. When the tibia is involved, it usually affects the proximal or middle third part of the bone. This pathology is most often seen in athletes or related to traumatic activities.

This case describes a healthy, pre-menopausal, non-athlete woman presenting with a distal tibial atraumatic stress fracture. Diagnosis is usually confirmed by a CT scan or MRI since radiographs could often show no abnormalities. Treatment of such fractures is conservative in the majority of cases and when present, predisposing or causative factors should also be investigated and assessed.

## Introduction

Stress fractures commonly affect the weight-bearing bones in the lower extremities, particularly the sacrum (29.6%), tibia (23.6%), neck of the femur (9.9%), tarsal navicular (17.6%), metatarsal bones (16.2%), and calcaneus (2.8%), and are typically unilateral in nature [1]. Stress fractures can be caused either by a normal bone subjected to an abnormal load of stress (fatigue fracture) or an abnormal bone with a normal stress load (insufficiency fracture). Both are caused by the bone being subjected to prolonged, cumulative, and low-intensity pressures [2]. Professional sports, military personnel, and ballet dancers have all reported developing occupational stress fractures with the tibia being the most typical site among athletes [3], metatarsal fractures in soldiers [4], and both tibial and fibular stress fractures in ballet dancers [5]. Genetic factors, hormonal abnormalities, metabolic bone disorders (such as osteoporosis and osteomalacia), liver and kidney failure, systemic diseases, rheumatic diseases, nutritional deficiencies, heavy smoking, and use of medications that may cause osteoporosis are all predisposing factors for stress fractures [6,7].

Stress fractures of the tibia secondary to sports-related activities are relatively common [2]. However, the rarity of this case resides in its atypical location at the distal tibia in a pre-menopausal woman with no history of trauma or secondary causes of osteoporosis.

## Case presentation

**Figure 1:**
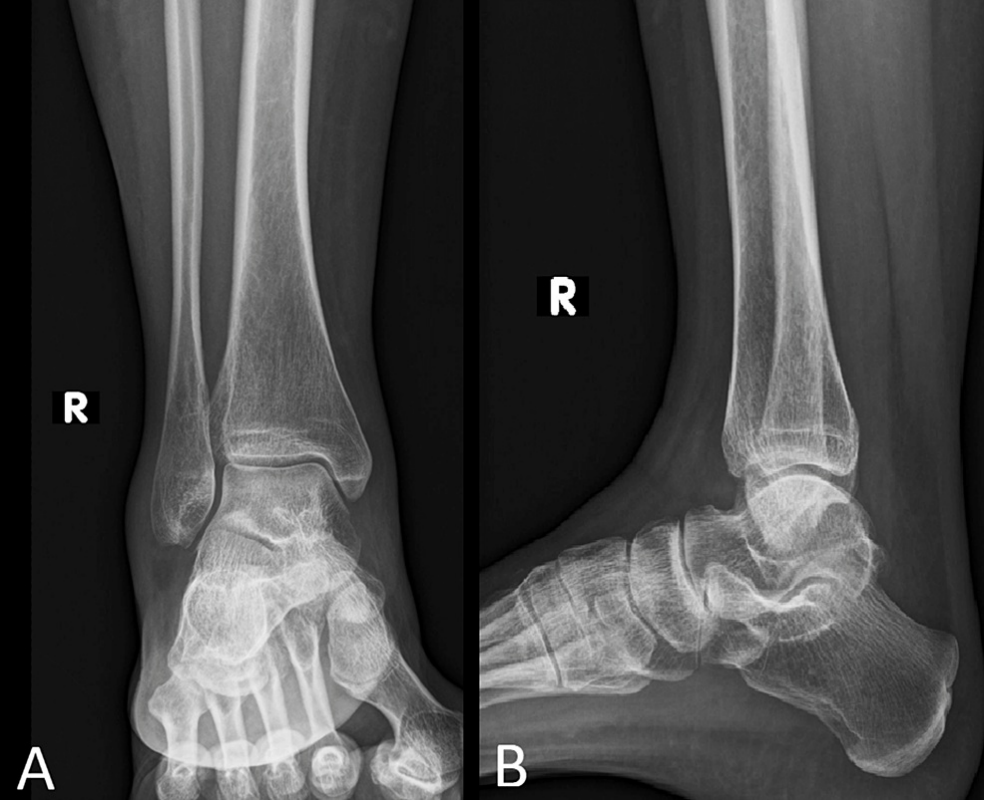
Plain radiographs of the right ankle (A) anteroposterior and (B) lateral views showing no bony abnormalities.

The patient was a 44-year-old female patient, pre-menopausal and previously healthy, presenting with a one-week history of atraumatic ankle pain with difficulty ambulating. She leads a sedentary lifestyle and does not regularly participate in any load-bearing type of sports. She has no family history of metabolic or connective tissue disorders. On physical exam, she was found to have mild edema, erythema, and point tenderness over the distal anterior aspect of the tibia with antalgic restrictions of range of motion especially upon dorsiflexion of the ankle, with moderate pain upon ambulation. No signs of neuropathy were present upon monofilament testing. Plain radiographs were done showing no bony abnormalities (Figure 1).

**Figure 2:**
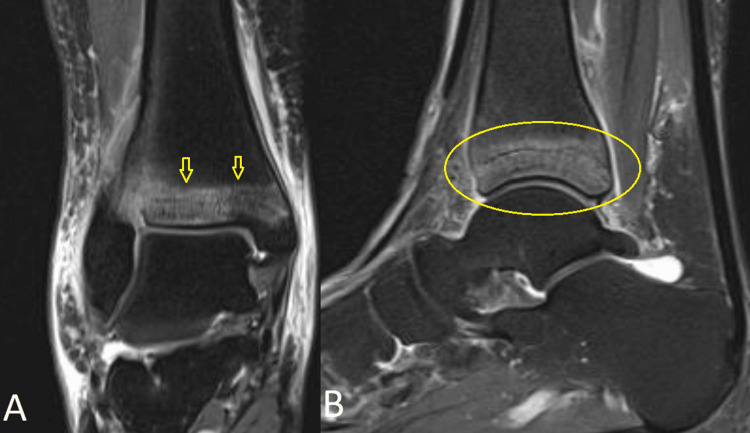
MRI of the right ankle in the (A) coronal and (B) sagittal fat saturated views showing an acute oblique non-displaced fracture of the distal tibial metaphysis with surrounding mild effusion of the subtalar and tibiotalar joints.

The patient was discharged on supportive management (weight-bearing being allowed) consisting of an ankle brace along with a course of anti-inflammatories with rest, and icing. One week later, she came back to the clinic with increasing pain over the ankle and difficulty ambulating. On physical exam, there was mild swelling of the ankle joint, no warmth with pain upon dorsi- and plantar flexion with no significant laxity that may suggest a syndesmotic injury. Blood workup consisting of complete blood count, inflammatory markers, glucose, A1C, and uric acid showing normal ranged results. Thus, an MRI was done for further assessment. MRI of the ankle showed a stress fracture of the distal tibia with periosteal, adjacent soft tissue edema, and band-like bone marrow edema at the metaphyseal area with extension of the fracture line to the subarticular surface contour of the distal tibia at the level of the tibiotalar joint (Figure 2).

Apart from the fracture, the bone structure of the entire tibia did not reveal any associated lesions. There were no factors in the history of the patient that could explain this fracture. The findings of routine laboratory tests, such as those for calcium, phosphorus, alkaline phosphatase, vitamin D, and parathyroid hormone, were all normal. In addition, thyroid functions, acute phase response proteins (such as C-reactive protein and erythrocyte sedimentation rate), and rheumatic profile were all within normal ranges.

The patient was treated conservatively with a posterior cast with a non-weight bearing on her affected leg. On six weeks follow-up, the posterior cast was removed. The patient reports no residual pain and no tenderness. She was started on a physical therapy protocol consisting of anti-inflammatory modalities, proprioceptive exercises, and weight-bearing as tolerated.

## Discussion

The earliest description of stress fractures is credited to Breithaupt, a Prussian military surgeon. In 1855, he made notice of the prevalence of "March fractures" in fresh military recruits. Currently, stress fractures are classified as either fatigue fractures more commonly seen in athletes, or insufficiency fractures usually occurring in postmenopausal osteoporotic women [8]. In the above-described case, the stress fracture occurred in a healthy, pre-menopausal woman without any history of sports or activity-related trauma.

The proximal or middle portion of the bone is where most tibial stress fractures take place [8]. Distal tibial stress fractures are a rarely described entity, mainly occurring in athletes, making this case unique and undescribed previously.

Radiography, computed tomography, and magnetic resonance imaging are all supplementary imaging techniques. The main benefit of radiographs in the initial assessment of the patient is in ruling out other potential causes of the patient's symptoms because they are somewhat insensitive for the detection of stress fractures [8]. In fact, they are initially inconclusive [9] but can later appear due to periosteal response and cortical thickening at the location of the injury, which gradually makes the fracture evident on plain radiographs [1]. This may delay the diagnosis ranging usually from 15 days to 18 months [7]. This was the case in the described patient. An MRI was done due to the persistence of pain since this imaging modality is the most sensitive modality for detecting stress fracture, and may also be useful for differentiating ligamentous and cartilaginous injury from a bony injury. On the bone scan, a characteristic diffuse abnormal activity may be seen involving the distal tibia in both fatigue and insufficiency fractures [6-9].

Periostitis, infections, tibial stress reaction, medial tibial stress syndrome, anterior compartment syndrome, tumors (such as osteoid osteoma), and metastases should all be considered in the differential diagnosis which makes the MRI an essential test for the diagnosis [6,9,10].

Rest, activity modification, and cast immobilization for a period of six to twelve weeks are the mainstays of treatment for stress fractures [7]. A combination of relative rest and non-invasive treatments is usually enough to control the majority of such fractures [9]. In certain situations with persistent or repeated fractures, surgical intervention including open fixation is one of the viable options [1]. Additionally, in case the stress fracture is secondary to a certain pathology, the latter must be addressed separately [7]. Our patient was pain-free and able to resume her activities of daily living after six weeks of conservative therapy.

## Conclusions

This is a rare case of a healthy, pre-menopausal woman presenting with a distal tibial stress fracture unrelated to physical activity or trauma. Usually, stress fractures occur in the proximal or middle third of the tibia and are most commonly related to sports. However, this case shows that this entity should be considered in every patient presenting with non-specific atraumatic ankle pain. X-rays usually reveal no abnormalities in the early stages. An MRI is most of the time necessary to eliminate other diagnoses. The treatment is usually conservative consisting of rest, activity modification, and cast immobilization. However, causative or predisposing factors should be managed separately when present.
